# Recent Progress in Unraveling Central Nervous System Processing of Itch Sensation

**DOI:** 10.1097/WOX.0b013e318187ff70

**Published:** 2008-10-15

**Authors:** Florian Pfab, Michael Valet, Thomas Tölle, Heidrun Behrendt, Johannes Ring, Ulf Darsow

**Affiliations:** 1 Department of Dermatology and Allergy, Technische Universität München, Technische Universität München, Biedersteiner Strasse 29, 80802 Munich, Germany; 2 Division of Environmental Dermatology and Allergy, Helmholtz Zentrum München/TUM, ZAUM-Center for Allergy and Environment, Technische Universität München, Biedersteiner Strasse 29, 80802 Munich, Germany; 3 Department of Neurology, Klinikum r.d. Isar, Technische Universität München, Munich, Germany

**Keywords:** itch, cerebral processing, neuroimaging, histamine

## Abstract

Itch is the major symptom of many allergic or inflammatory skin diseases, yet it is still difficult to measure objectively. This article shows and updates the development and approaches of central nervous system investigation of itch.

Human neuroimaging studies on the physiology and pathophysiology of itch sensation have been hampered by the lack of a reproducible "on-off" stimulus. Short-term alternating temperature modulation of histamine-induced itch has recently been shown to provide on-off characteristics.

Recent studies with functional magnetic resonance imaging demonstrate that itch sensation in healthy volunteers is processed by a network of brain regions contributing to the encoding of sensory, emotional, attentional, evaluative, and motivational aspects of itch.

## 

The sensation of itch was already defined in the 17th century as a complex and unpleasant sensory experience that induces the urge to scratch[1]. It is a crucial symptom of inflammatory skin diseases [2,3] and difficult to be measured objectively. With its well-known psychophysiological aspects, it has substantial impact on the quality of life of patients[4] Its pathophysiology remains poorly understood despite numerous studies[2].

Itch can easily be elicited experimentally--most effectively via a histamine stimulus[5]. With its mainly subjective characteristics, itch has some psychophysiological similarity to pain. Although some degree of overlap is present, recent neurophysiological studies have confirmed that itch pathways are clearly distinct from pain pathways[6-8]. In recent years, progress in central nervous system imaging technologies had substantial impact on itch research. New models to measure itch may also be useful for the development of new therapeutic strategies against pruritus.

## Eppendorf itch questionnaire

The quantity and quality of perceived itch show specific characteristics in different pruritic skin diseases. Clinical observations point to differences in the central nervous processing of pruritus. The multidimensional Eppendorf Itch Questionnaire (EIQ) [9] was used in hospitalized patients experiencing atopic eczema (AE, n = 62) or chronic urticaria (n = 58). Total scores (127 items), emotional and sensory ratings, reactive behavior and visual analogue scale (VAS) ratings for itch intensity were evaluated. The mean VAS ratings of itch intensity showed no significant difference between the two diseases. In contrast, the total EIQ score was significantly higher in the AE group with 231.6 ± 11.5 versus 175.2 ± 9.4. In 34 of 127 items, a significantly different rating was obtained, mostly with higher load for affective and some sensory items in AE. Significant differences were also seen in the description of the scratch response. Thus, itch perception in AE and chronic urticaria differs on a qualitative level, influencing items relevant for quality of life. Similar findings were perceived in a study investigating the preventive effect of acupuncture on experimental itch, showing a preventive point-specific effect on emotional items of the EIQ[10]. These findings accentuate the emotional component of the itch sensation with possible differences in CNS processing.

## First neuroimaging studies about itch using positron emission tomography

One of the first neuroimaging studies investigating the cerebral processing of itch correlated subjective itch sensation with central activation: a complex pattern of cerebral activation after experimental itch induction (skin prick model) with histamine dihydrochloride (0.03%-8%) at the right lower arm in healthy volunteers was observed in a H_2_[15]O positron emission tomography (PET) correlation study (n = 6)[6]. Subtraction analysis of histamine versus control condition revealed significant activation of the primary sensory cortex and motor-associated areas, predominantly left-sided activations of frontal, orbitofrontal, and superior temporal cortex and anterior cingulate cortex. Compared with activation patterns induced by pain stimuli[11], itch did not lead to thalamus activation, but significant activation in the insula region and differences in sensory, motor, and cingulate areas. Hsieh et al[12] reported similar findings already in 1994, giving first evidence for central nervous processing of itch and clearly demonstrating differences to pain processing. Planning of a scratch response is mirrored by extensive activation of motor areas in the cortex; other areas may be involved in emotional evaluation of pruriception.

## New methodology provides a phasic stimulus

The reason for the lack of functional magnetic resonance imaging (fMRI) studies on itch until recently was the nonexistence of a phasic stimulus. In contrast to pain, no method had been described to increase and decrease the sensation of itch within seconds. A new approach gives a recent psychophysical study, where itch sensation was investigated using a methodology with short-term temperature changes for modulation of histamine-induced itch[13].

In 9 healthy right-handed male volunteers (mean age, 29 ± 2.6 years), 1% histamine dihydrochloride was used in the skin prick model as standard itch stimulus on the right forearm with subsequent thermal modulation of the target skin area using a Medoc TSA II NeuroSensory Analyzer thermode. Modulation occurred in rapid alternating order from 32°C (neutral block, 20 seconds) to 25°C (slightly cold block, 20 seconds) and vice versa, 14 times in series.

Subjective itch ratings were recorded using a computerized VAS ranging from 0 to 100 at 4-second intervals.

**Figure 1:**
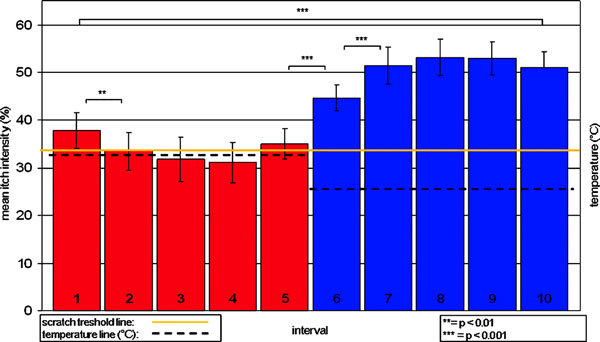
**Biphasic stimulus model using 32°C stimulation to decrease and 25°C stimulation to increase histamine-induced itch**. The red/blue columns represent the mean itch intensity (VAS range, 0-100) of 9 healthy volunteers obtained every 4 seconds during the 20 seconds of the 32°C stimulation (red columns were numbered 1-5) and during the 20 seconds of the 25°C stimulation period (blue columns were numbered 6-10). Two sessions, each with 14 consecutive cycles of 32°C followed by 25°C stimulation, were applied. The yellow line represents the scratch threshold (33% itch intensity). Asterisks indicate significant differences between rating intervals. **P < 0.01, ***P < 0.001. From Pfab et al[13].

All subjects reported localized itch sensations without pain. In each individual subject as well as in the total group, significant differences between VAS rating intervals concerning itch intensity were noted. Itch intensity was generally perceived as higher during 25°C blocks than during 32°C blocks. Mean itch intensity was 50.6% ± 3.5% during the 25°C block (intervals 6-10) and 33.8% ± 3.9% during the 32°C block (intervals 1-5) with a highly significant difference (P < 0.0001) between the 2 temperature blocks (Figure 1).

Despite the common knowledge that intensive cold inhibits itch sensation, a reproducible significant enhancement of histamine-induced itch by short-term moderate cooling was shown. This effect might be explained by peripheral and central adaptation processes triggered by abnormal afferent activity patterns.

This method allows controlled and rapid modulation of itch. Short-term cooling enhances histamine-induced itch, providing the possibility of further and more detailed investigations of itch by functional imaging methods, such as fMRI.

## First fMRI Study using Short-term Alternating Temperature Modulation

Using the previously established biphasic temperature stimulus model, we investigated the cerebral activation pattern of itch processing in 12 healthy volunteers with fMRI[14].

Itch was provoked on the right forearm with 1% histamine dihydrochloride. Local temperature modulation allowed reproducible itch provocation above scratch threshold (defined as 33/100 on a VAS) during 25°C, whereas itch declined below scratch threshold during the 32°C stimulation period. No itch sensation was reported using 0.9% saline with temperature modulation.

**Figure 2:**
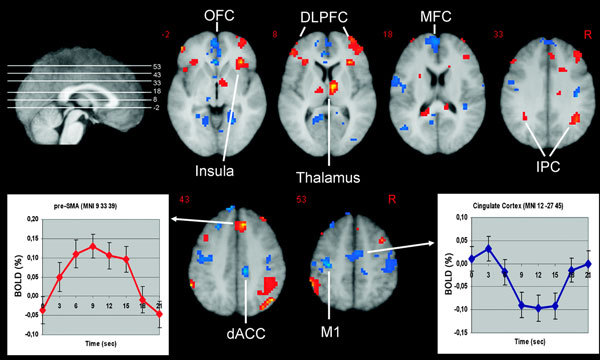
**The increase of histamine-induced itch during the first 8 seconds of the 25°C stimulation periods (as compared with saline) is associated with an increase (red) and decrease (blue) of activation in various brain structures subserving sensory, emotional, cognitive, and motivational aspects of itch processing**. As an example, the averaged relative fMRI BOLD signal of all subjects during 20 seconds of the 25°C stimulation period is presented: for the pre-SMA region with increased and the cingulate cortex with decreased activation in comparison with saline. DLPFC indicates dorsolateral prefrontal cortex; IPC, inferior parietal cortex; MFC, medial frontal cortex; OFC, orbitofrontal cortex; R, right side of the brain. From Valet et al[14].

The calculation of itch-specific activation maps for the first 4, 8, 12, 16, and 20 seconds of the 25°C stimulation period confirmed that the changes during the first 8 seconds are reflected by the highest brain activations during this period than during the other period. Focusing on the first 8 seconds of 25°C stimulation, the thalamus, presupplementary motor area (pre-SMA), lateral prefrontal cortex, anterior insular cortex, and inferior parietal cortex were more active than during the saline condition (P < 0.001; Figure 2). The medial frontal cortex, the orbitofrontal cortex, the dorsal part of the anterior cingulate cortex (dACC) and the primary motor cortex (M1) were less active during histamine-induced itch than during saline (P < 0.001; Figure 2).

So far, this is the only imaging study on itch using a phasic suprathreshold itch model comparing itch and non-itch phases.

## Other neuroimaging studies

Neuroimaging studies on itch have been done using [15O]H2O-PET[6,12,15,16,20] and, more recently, fMRI[17-21]. Table 1 summarizes their findings.

**Table 1:** Summarizing Findings of Current Neuroimaging Studies

	Healthy Volunteers									Atopic Patients	
	
	**Hsieh et al**[12]**PET**	**Drzezga et al**[15]**PET**	**Mochizuki et al**[16]**PET**	**Schneider et al**[20]**PET**	**Walter et al**[17]**fMRI**	**Leknes et al**[19]**fMRI**	**Valet et al**[14]**fMRI**	**Mochizuki et al**[18]**fMRI**	**Herde et al**[22]**fMRI**	**Leknes et al**[19]**fMRI**	**Schneider et al**[20]**PET**
Neuroimaging analysis	Subtraction	Correlation	Subtraction	Correlation	Correlation	Correlation	Subtraction	Correlation	Subtraction	Correlation	Correlation
Itch induction	Intracutaneous injection	Skin prick model	Iontophoresis	Iontophoresis	Skin prick model	Skin prick model	Skin prick model	Iontophoresis	Intracutaneous microdialysis	Skin prick model	Iontophoresis
Itch stimulus	Histamine	Histamine	Histamine	Histamine	Histamine	Histamine	Histamine	Histamine	Histamine and codeine	Histamine	Histamine
Brain region	--	--	--	--	--	--	--	--	--	--	--
Primary somatosensory cortex (BA 1-3)	--	I, C	--	C	--	--	C-	--	C	C	--
Somatosensory association/parietal cortex (BA 5, 7)	--	I, C	I	C	--	--	--	--	C	--	--
Primary motor cortex (BA 4)	--	C	--	--	--	--	--	--	--	C	--
Premotor and presupplementary motor cortex (BA 6)	I, C	I, C	I	C	--	--	I	I	I, C	C	I, C
Cerebellum	I, C	--	--	--	C	--	--	--	I, C	--	I, C
Insular cortex (BA 13, 14)	--	C	--	--	--	I, C	C	C	I, C	--	I
Cingulate cortex (BA 23, 24, 25, 32)	C	I, C	C	C	C	I, C	I-	C	I, C	I, C	--
Prefrontal cortex (BA 9)	I, C	C	--	I	--	--	--	--	I, C	--	I
Frontopolar and orbitofrontal area (BA 10-12)	--	C	--	--	I, C	--	I-, C-	I	C	I, C	I
Inferior parietal and dorsolateral prefrontal cortex (BA 45, 46)	--	C	I, C	--	--	--	I, C	--	I, C	--	--
Temporal gyrus (BA 20-22)	--	--	--	--	C	--	--	--	I, C	--	--
Temporal lobe/Wernicke area (BA 38-40)	--	--	I	--	--	--	I, C	--	I, C	C	C
Thalamus	--	--	C	--	--	I, C	I, C	--	I, C	I, C	C
Basal ganglia	--	--	--	--	--	I	--	I	I, C	I, C	I

Activation in the corresponding region is marked by I (ipsilateral) or C (contralateral); the minus sign (-) indicates reduced activation in comparison with a saline control stimulus.

It is important to take the different methods of itch provocation (superficial intracutaneous application of histamine) into consideration and their study design regarding correlation of subjective itch ratings with brain activation[17,19].

Studies using intracutaneous histamine injection[12] or especially intracutaneous microdialysis[22], which are capable of inducing pain might have influenced itch processing by an overlap of pain. A histamine prick model as previously described, which was used in other studies[6,14,15,17,19], does not provoke sensations of pain[5].

The approach of correlation analyses of subjective itch ratings with the fMRI blood oxygenation level-dependent (BOLD) signal[17,19] to detect itch-related brain activations is another methodological concern: continuously obtaining itch ratings during fMRI scanning might induce motor and cognitive interactions confounding itch-related imaging results.

In contrast to the aforementioned studies investigating the neuronal effects of pruritus, a recently published fMRI study focused on another aspect, namely scratching, which is an often seen behavioral response to pruritus[21]. However, a limitation of the study might be that the sensory effects of scratching without any induction of itch were measured.

So far, 2 studies used a stimulus method that achieved strong repeatable pruritus comparing itch and non-itch periods within seconds[14] or minutes[22].

Taking the results of these studies together, cerebral key regions involved in the perception and processing of histamine-induced itch in healthy volunteers seem to be the premotor area and pre-SMA, the cingulate and insular cortex, thalamus, and prefrontal cortex (inferior and dorsolateral prefrontal cortex).

So far, one study[19] investigated the effect of allergen-induced itch in patients with mucosal atopy showing similarities to histamine-induced itch (Table 1). Mochizuki et al[18] directly compared itch with pain stimuli using fMRI in healthy volunteers. Neural activation in the posterior cingulate cortex and the posterior insula was significantly higher during itch than during pain. Pain in contrast to itch induced an activation of the thalamus correlating to subjective pain sensation.

## Important brain regions involved in itch processing and their functions

### Pre-SMA and primary motor cortex, motor part of the cingulate cortex

The pre-SMA is thought to encode motor actions before self-initiated voluntary movements and during imagination of motor action[23]. Primary motor cortex is typically involved in motor planning and execution, highlighting the definition of itch that includes the intention to scratch[1]. As the subjects were not allowed to scratch, the deactivation observed in our fMRI study might indicate a suppression of motor activity. The dACC is also thought to be engaged in premotor planning[24,25], as well as in stimulus intensity encoding[26,27]. Translating this information from pain to itch processing, we hypothesize that the dual function of the dACC and the anatomical neighboring to M1 is advantageous for the generation of an adequate motor response to the itching stimulus (planning of a scratch response) in relation to the processed sensory information.

### Inferior parietal cortex and dorsolateral prefrontal cortex

The inferior parietal cortex is known to be involved in the spatial representation of the intrapersonal and extrapersonal space (body scheme) and regarded as polymodal association area, integrating multisensory information from the thalamus, insula, anterior cingulate cortex, and prefrontal cortex[28]. It is known that lesions of this region in the nondominant hemisphere are highly associated with neglect and inattention syndromes. Activation of this region may therefore reflect a spatially directed attention to the itching stimulus.

The dorsolateral prefrontal cortex is associated with cognitive evaluative, attention-dependent, working memory, and executive functions[29]. Besides the input from the thalamus and cingulate cortex, it receives and processes multisensory information mainly from the inferior parietal cortex[29]. The sensory convergence and integration is required in the preparation of motor action.

### Insular cortex

The anterior insula is assumed to subserve subjective feelings[30] and to integrate sensory and emotional experiences[31]. It has been suggested that the insular cortex is part of an interoceptive system providing the basis for a cortical image of homeostatic activity that reflects all aspects of the physiological condition[32]. In this context, the activation of the insular cortex might indicate an interference on the homeostatic balance by the sensation of itch, leading to the desire to scratch[13,19].

### Thalamus and primary somatosensory cortex

The activation of the thalamus and primary somatosensory cortex (S1) can be attributed to sensory aspects of itch processing. The ability to locate itch plays an important role in the initiation of withdrawal behavior. These brain structures are fulfilling important functions regarding detection, localization, discrimination, and intensity encoding of sensory stimuli[33].

## Conclusions

The itch sensation is processed by a network of brain regions contributing to the encoding of sensory, emotional, attention-dependent, cognitive-evaluative, and motivational patterns. It now seems possible to further analyze the specific effects of various therapies on these significant activation patterns.
